# Bovine Genome Database: supporting community annotation and analysis of the *Bos taurus *genome

**DOI:** 10.1186/1471-2164-11-645

**Published:** 2010-11-19

**Authors:** Justin T Reese, Christopher P Childers, Jaideep P Sundaram, C Michael Dickens, Kevin L Childs, Donald C Vile, Christine G Elsik

**Affiliations:** 1 Department of Animal Science, Texas A&M University, College Station, TX 77843 USA; 2 Department of Biology, Georgetown University, Washington, DC 20057 USA; 3 Department of Plant Biology, Michigan State University, 166 Plant Biology Building, East Lansing, MI 48824, USA

## Abstract

**Background:**

A goal of the Bovine Genome Database (BGD; http://BovineGenome.org) has been to support the Bovine Genome Sequencing and Analysis Consortium (BGSAC) in the annotation and analysis of the bovine genome. We were faced with several challenges, including the need to maintain consistent quality despite diversity in annotation expertise in the research community, the need to maintain consistent data formats, and the need to minimize the potential duplication of annotation effort. With new sequencing technologies allowing many more eukaryotic genomes to be sequenced, the demand for collaborative annotation is likely to increase. Here we present our approach, challenges and solutions facilitating a large distributed annotation project.

**Results and Discussion:**

BGD has provided annotation tools that supported 147 members of the BGSAC in contributing 3,871 gene models over a fifteen-week period, and these annotations have been integrated into the bovine Official Gene Set. Our approach has been to provide an annotation system, which includes a BLAST site, multiple genome browsers, an annotation portal, and the Apollo Annotation Editor configured to connect directly to our Chado database. In addition to implementing and integrating components of the annotation system, we have performed computational analyses to create gene evidence tracks and a consensus gene set, which can be viewed on individual gene pages at BGD.

**Conclusions:**

We have provided annotation tools that alleviate challenges associated with distributed annotation. Our system provides a consistent set of data to all annotators and eliminates the need for annotators to format data. Involving the bovine research community in genome annotation has allowed us to leverage expertise in various areas of bovine biology to provide biological insight into the genome sequence.

## Background

The Bovine Genome Sequencing and Analysis Consortium (BGSAC) carried out one of the largest distributed annotation projects for a eukaryotic genome. A goal of the Bovine Genome Database (BGD; http://BovineGenome.org) has been to support the Bovine Genome Sequencing and Analysis Consortium (BGSAC) in the annotation and analysis of the bovine genome. The central data for BGD are assemblies of the *Bos taurus *genome, which was sequenced to 7.1 fold coverage [1]. BGD currently provides data for assemblies Btau_3.1 and Btau_4.0 [2], which were generated by the Baylor College of Medicine Human Genome Sequencing Center (BCM-HGSC), although alternative assemblies are available elsewhere [3]. Since our initial goal was to support the activities of BGSAC, our effort has been focused on tools and datasets to facilitate annotation and to maintain organization to reduce duplicated efforts.

As the project started, we faced a challenge that is typical for many new genome projects: the sequencing data was available before the genome database and annotation tools were ready for the eager annotators. As a result, we were forced to deploy applications without rigorous testing, and when bugs were reported, we had to modify the live site. The rise of many new genome projects due to new, cost-effective sequencing technologies, will lead to an increased demand for annotation tools that novices can use. Here we present our approaches, challenges and solutions with the aim of informing future genome principal investigators, who may have limited experience in genome annotation or database development.

## Construction and Content

### Genome Browsers

We began developing BGD upon release of the bovine genome assembly Btau_2.0. Btau_2.0 was the first bovine assembly in which contigs were assembled in scaffolds. Our first step was to set up a GBrowse genome browser with a MySQL database serving as the backend [4]. GBrowse allows for simultaneous viewing of all data sets associated with a particular region of the genome. Although it was premature to annotate Btau_2.0, we presented the BGD GBrowse at several international conferences to create interest and attract potential annotators from the research community.

BGD now includes GBrowse sites for the newer assemblies, Btau_3.1 and Btau_4.0. BGD maintains GBrowse sites for each assembly on scaffold or chromosome coordinate systems. Each chromosome-coordinate-based GBrowse has a track showing ordered scaffolds, with links to the corresponding scaffolds in the scaffold-coordinate-based GBrowse. Although the GMOD Chado schema [5] is compatible with GBrowse and setting up a Chado database was our next step, we chose to maintain separate MySQL databases for each implementation of GBrowse to improve query performance. The MySQL databases are routinely synchronized with the Chado PostgreSQL databases.

### Genome Database and Community Annotation System

BGD relies heavily on software produced by the GMOD project [6]. In addition to the GBrowse and Chado database schema, we have incorporated the Apollo Annotation Editor [7], and XORT and GMODTools for bulk data exchange in and out of our Chado database, respectively. We employ the PostgreSQL database management system structured with the Chado schema for the complete sets of assembly and genomic feature data. Chado uses controlled vocabulary (CV) terms from the Sequence Ontology (SO) [8]. Although Chado was originally designed for use by FlyBase [9], it has since been deployed by several other model organism databases.

Before BGD was developed, curators at FlyBase had been the primary users of the Apollo annotation software, and data exchange between annotators and the database occurred only through flat XML files. We were the first research group to implement the Apollo system for the annotation of a mammalian genome. An initial concern was the large size of genes in mammals, due to long introns. For any one gene, Apollo would be required to load a longer segment of chromosome and hold more data in memory, potentially a challenge for personal computers with little memory. To minimize the amount of required memory, we chose to develop the tools to annotate features on scaffolds instead of whole chromosomes.

Our first Chado database contained data from Btau_3.1, the assembly that the BGSAC used for annotation. An independent database was later developed for Btau_4.0. One of our motivations for using Chado has been the feasibility of directly sending data to remote Apollo software clients on users' workstations to help with annotation. Early in the development of this community annotation system we encountered technical challenges, such as identifying SO terms for computed gene models that were compatible between Chado and Apollo. Discontinuity of funding for Apollo resulted in discrepancies between CV terms used by Apollo and those used by Chado and other GMOD components. For example, many GMOD components load protein features into Chado using the term "protein", but the SO term is "polypeptide". We approached this SO mismatch problem by trial and error: creating generic feature format (GFF) files using different SO terms, loading the GFF into the database, and checking whether or not the track was displayed correctly in Apollo. Renewed support for Apollo has currently mitigated most of the issues we initially encountered. We have attained Chado-Apollo compatibility by modeling computational gene data as a three-tiered hierarchy, with the features of CV term type "gene" at the highest level of the hierarchy. Each "gene" feature has one or more features of CV term type "mRNA" and each "mRNA" feature in turn has one or more features of CV term type "exon", as well as one feature of CV term "protein". We have also modified the way exons and mRNA features are modeled. One way to minimize the size of the Chado database is to allow mRNA features to share exons when appropriate. However, we discovered errors when dumping FASTA formatted sequences using GMODTools, so we have not allowed exon features to be shared between different mRNA features.

Our system allows experts in bovine biology to contribute to annotation of the bovine genome using the Apollo Annotation Editor, installed on users' desktops. The system is composed of the main Chado database, an intermediate Chado database to hold pre-reviewed submissions, the Apollo Annotation Editor, an Apollo-Chado adapter (included in the Apollo package), a community annotation web portal for user authentication and coordination of annotation efforts, XORT (a component of GMOD) to load manual annotations in Chado-XML format into the Chado databases, and GMODTools to dump annotation data from Chado. Although we initially developed the annotation system for Btau_3.1, we now only support annotation of Btau_4.0. Routine maintenance on the Chado PostgreSQL databases includes running postgres vacuum after data is loaded (which greatly improves database performance in our hands), and regular database backups using the PostGreSQL command pg_dump.

Because default configuration files were designed for FlyBase, Apollo must be configured for each project separately. We have modified the chado-adaptor.xml file to provide Apollo with connection information for our server and Chado database, and to describe the data available in our database. We have also created the bovine.tiers file, which defines the different data tracks available and also allows for additional settings to control how the data are displayed. The tiers file also allows incorporation of URLs for features in the gene evidence tracks, allowing users to obtain more information about the features. In BGD, the homolog alignment tracks are linked to their source webpage at NCBI, Ensembl or Uniprot. Ab initio gene predictions and consensus gene models are linked to data on the BGD server via CGI scripts. The bovine.style file contains the name of the tiers file and the organism name for the species to be annotated. In addition to these settings, the bovine.style file also contains a series of pre-generated comments that can be used by annotators to describe gene models. These "canned comments" have been useful for standardizing annotation notes, and have been customized for the bovine annotation project. We have also updated the Apollo chado.style and apollo.cfg files to include the style information for the new species.

Our community annotation web portal consists of a set of CGI scripts to authenticate users and accept uploaded annotations, as well as a MySQL database that maintains user information and serves as a back-end for annotation query web pages. The portal has allowed users to register, login, download Apollo software configuration files and tutorials, and sign up to annotate priority genes. Annotation submission pages have allowed users to either upload Chado-XML files exported from Apollo or enter annotation information manually into web forms. Users can search for submitted annotations by user name or user-submitted information, such as gene name, gene family or keywords. Users can also view all submissions and edit their own submissions.

### Data Exchange During Initial Annotation of the Bovine Genome

**Figure 1:**
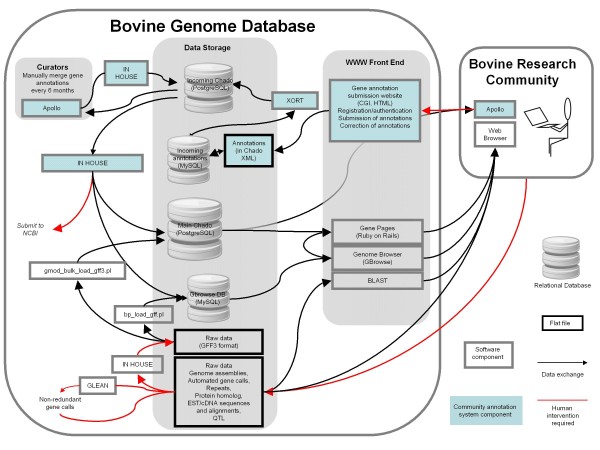
**Exchange of data within BGD and between BGD and the bovine research community during the BGSAC annotation project **[1]. Red lines indicate steps that require human intervention. Gray lines indicate steps that do not require human intervention.

During the BGSAC annotation project, data was exchanged within BGD and with the bovine research community as described in Figure 1. Computational results were formatted into GFF3 and loaded using XORT into the Chado PostgreSQL database. In addition, a subset of the GFF3 was loaded into the Gbrowse MySQL databases. The Chado PostgreSQL database supplied data to the Gene Pages and to the Apollo annotation editor.

Annotators accessed computational gene evidence by starting Apollo and entering the following information in the startup menu of the Apollo client software: 1) the BGD server hostname and 2) either an OGS identifier for a gene of interest or a scaffold identifier and coordinates designating the region of interest. Apollo then accessed the assembly and gene feature data from the Chado PostgreSQL database for the specified region. The annotator edited existing gene models or created new gene models and saved the results in Chado-XML format using the File pull-down menu in Apollo. The annotator then logged in to the BGD community annotation portal and uploaded the Chado-XML file to the BGD server. The uploaded files were saved in a secure directory on the BGD server, with the user id and a timestamp appended to the filename. Upon upload, the Chado-XML file was processed by a Perl script which used the Perl DBI module to load the information into the annotation portal MySQL database so that the data would be immediately visible on the annotation portal website, with a temporary id consisting of the user id and automatically incremented digits. Periodically, a BGD curator used XORT to load the Chado-XML files into the intermediate Chado database for pre-reviewed annotations. The curator then used GMODTools to retrieve the annotations from the Chado database as GFF3 and FASTA sequence files. The curator first performed automated checks on the GFF3 and FASTA coding sequences to flag potential conflicting annotations and coding sequences that have stop codons for further inspection and revision. The checked manual annotations were loaded into the main Chado database after being assigned BGD identifiers and incorporated into a new release of the OGS.

### Data Exchange in the Next-generation BGD

**Figure 2:**
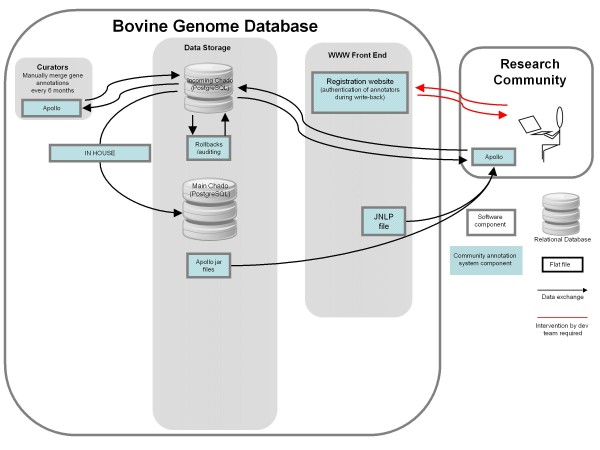
**Improved BGD system for ongoing annotation of the bovine genome**. The new features are 1) support for direct writebacks from the user's Apollo client to the intermediate Chado database, 2) support for launching and installing Apollo using Java WebStart, and 3) support for database auditing and rollbacks. Other components (e.g. genome browser, gene pages, BLAST site) are the same as those shown in Figure 1, and are not shown here. Red lines indicate steps that require human intervention. Gray lines indicate steps that do not require human intervention.

We have made several improvements to the annotation system after the BGSAC annotation project and publication of the bovine genome (Fig 2), including support for direct writebacks from Apollo to the intermediate Chado database, database auditing and support for installing and launching Apollo using Java Webstart. Support for direct database writebacks allows users to upload their annotations directly from Apollo to intermeduate Chado database. This increases annotator efficiency and improves user experience by eliminating the need to save and upload Chado-XML files. It also allows users to view the work of other annotators, which greatly reduces redundancy. To support database auditing and rollbacks, we extended the Chado schema to include an audit module, composed of several tables and database triggers. These triggers record any changes in the database to the audit module tables, allowing BGD administrators to monitor annotator activity. In the future, the audit module will also allow BGD administrators to rolled back changes. Support for Java Webstart allows users to install, configure and open Apollo by simply clicking a hyperlink to a Java Network Launching Protocol (.jnlp) file on the BGD website. This eliminates the need for annotators to manually install and configure Apollo. Every time Apollo-Webstart is launched by a user, it checks BGD servers for updated Apollo Java Archive (.jar) files, and updates the user's Apollo configuration files (e.g. chado-adapter.xml, apollo.cfg, bovine.tiers) even after the user has installed Apollo.

### Gene Pages

To display details for each OGS gene, we deployed a novel web application based on Chado on Rails [10], a framework for developing web applications that use Chado databases. Data for any OGS gene of interest are retrieved from Chado using the Ruby-on-Rails (RoR) application, which follows the "model-view-controller" (MVC) pattern. The model component is composed of an object-relational mapping of the Chado database schema in RoR, allowing tables in the Chado database to be manipulated using Rails objects. The controller component provides the logic to retrieve genes and their associated data, and the view components provide the html templates to display the data retrieved from Chado. In addition to providing pre-computed information about genes, each gene page contains a link to a wiki page on which research community members can enter information about genes, associated literature, and suggestions for correcting gene models.

### BLAST

BGD features a website for BLAST [11] similarity searches implemented using the NCBI standalone WWW BLAST server software. BGD allows BLAST searching of twenty-six different sequence databases, including the genome assembly on scaffolds and chromosome coordinate systems, the Official Gene Sets, and each set of automated gene predictions. We have modified the BLAST output page to provide each hit identifier with a hyperlink to the corresponding sequence information and each hit alignment with a hyperlink to the corresponding genomic region in GBrowse, where a track for the alignment is displayed alongside the other evidence tracks in the region. To accomplish this, we created a CGI script that reads and reformats BLAST output. Hits to OGSv2 protein or coding DNA sequence records are linked to the gene page record for the relevant OGSv2 gene model. Other hits are linked to a sequence at an external database, such as GenBank, GenPept, Ensembl, or to sequence data from the BGD Chado database (for OGS, *ab initio *and GLEAN gene models).

To display BLAST hits to the assembly in GBrowse dynamically, we have leveraged the built-in distributed annotation system (DAS) [4] feature of GBrowse. Our CGI script creates a DAS track using HSP coordinates in the BLAST output and submits the track to GBrowse. The track is displayed as an "External Annotation Track" labeled with an arbitrary numerical identifier. The track is maintained for the user in browser cookies so that results of multiple BLAST searches may be accumulated and viewed simultaneously. Using built-in GBrowse functionality, the user also can edit and download tab-delimited coordinate files for each DAS track.

### Content Management

BGD uses Drupal [12], an open source content management system for web pages. All content is stored in a MySQL database and each block of content (e.g. a home page, sidebar or banner) is referred to as a node. This allows web content in the forms of nodes to be edited and combined in a modular way. Drupal offers many advantages over static HTML pages, including easier site editing and maintenance and easy theme creation and adjustment. Drupal's modular system has a required set of core modules and additional optional modules that may be used to expand functionality on the site. The large user base frequently contributes new modules and themes back to the Drupal website. Once downloaded, new themes and modules can be quickly enabled using the web-based interface.

BGD has used several modules to extend the capabilities of the base Drupal installation. The FCKeditor [13] is a WYSIWYG text editor that simplifies formatting content. The IMCE module [14] is a file upload/browser module that, when integrated with FCKeditor, makes it very simple to upload images and quickly add them to pages using a GUI interface. The Content Construction Kit (CCK) [15]is a set of modules that enables the addition of custom fields to nodes. For example, BGD has a custom content type called "News" which is used for news items. The CCK Views module can be used to create a special type of node called a block that only displays content tagged as News. This block is used to display the news items on the front page, in addition to the content on the front page. The InsertFrame module [16] extends on the HTML iFrame tag by pre-calculating the page height and automatically setting it for the displayed page. These Drupal modules have offered a mechanism to seamlessly integrate CGI scripts and other dynamically generated content into the BGD theme without additional coding. Drupal site themes are divided into two sections: one or more cascading style sheets (CSS), and PHP templates. Style sheets specify everything from header colors and font styles to the characteristics of the menu items. Pages are rendered from the PHP templates, which contain the logic for the organization and display of the node's content. This allows different content to be displayed on a page based on predefined conditions, such as whether a user is logged in as an administrator, collaborator, or guest. Simply editing the CSS and template files can therefore change an entire site's appearance, eliminating the need to make page-by-page modifications.

## Data Acquisition and Analysis

In addition to providing annotation tools, we performed computations to provide additional evidence to facilitate the annotation. We used GLEAN [17] to combine the various sets of gene prediction performed on Btau_3.1 into a consensus gene set, as described in [1]. The GLEAN consensus gene set became the bovine Official Gene Set version 1 (OGSv1). OGSv1 and manual annotations from Btau_3.1 were mapped to Btau_4.0 using GMAP [18] to facilitate the analysis published in [1]. We later created OGSv2 for Btau_4.0 using a combination of new gene sets generated on Btau_4.0 by GLEAN, RefSeq, and Ensembl, as well as the mapped manual annotations. BGD currently maintains datasets and tools for both OGSv1/Btau_3.1 and OGSv2/Btau_4.0 to allow users to compare assemblies. BGD has maintained automated protein coding gene prediction sets provided by others (described in [1]), including gene sets generated by Fgenesh, Fgenesh++ [19,20], GENEID [21], SGP2 [22], Ensembl [23,24] and RefSeq [24]. BGD has also incorporated bovine predicted pseudogenes from Ensembl and RefSeq, bovine non-coding RNA from Ensembl, bovine expressed sequence tags (EST) from dbEST [25], full-length bovine cDNA sequences from GenBank [26], and protein homologs from SwissProt [27], Ensembl and RefSeq. Bovine EST and cDNA sequences were aligned to the genome assemblies using GMAP [18]. Protein homologs were aligned using Exonerate [28]. Functional descriptions and gene names for OGSv2 genes were transferred from gene models produced by RefSeq and Ensembl, with criteria for the gene locus in question that 1) Ensembl or RefSeq coding sequence coordinates must overlap OGSv2 coding sequence coordinates, and 2) the relationship between the OGSv2 and Ensembl or RefSeq gene locus must not include a split nor merged gene model. For OGSv2 genes that overlap RefSeq gene models, GO annotations and gene symbols are automatically obtained on a weekly basis from NCBI for genes that overlap RefSeq gene model. If an OGSv2 gene does not overlap any RefSeq gene models, GO annotations are obtained for gene models that overlap Ensembl gene models using EnsMart [29]. In addition to computed functional annotations, BGD has maintained descriptions and gene names provided by the research community. Non-gene-centric data in BGD includes repeats, single nucleotide polymorphisms (SNPs), quantitative trait loci (QTL) data curated from literature and haplotype data.

## Discussion

Our experience in a previous annotation project [30] revealed challenges in collecting and organizing community annotation data. These challenges included 1) maintaining consistent quality despite the diversity in annotation expertise in the community, 2) maintaining consistent data formats and 3) minimizing the potential for duplicate annotations. Our Apollo-Chado approach addressed many of the issues related to annotation quality and data formats. We reduced the duplication of effort using a community annotation web portal that allows annotators to sign up for annotating particular genes and also presents annotators with a list of priority genes that the community wishes to have annotated.

There are several advantages of using Apollo with a direct connection to the Chado database. For example, from a user's standpoint, 1) users have easy access to pre-computed gene evidence; 2) Apollo provides a rich set of tools for viewing and editing gene models; and 3) there is no need to manually record sequences or coordinate information. With built-in splice site modeling and the view of multiple evidence tracks, Apollo provides immense improvement over the simplistic approach of copying and pasting results from a single BLAST alignment. In addition to providing tools that reduce annotator error, the Apollo-Chado approach provides consistency in the quality of gene evidence data used by all research community members. The effort required to organize community annotation data is significantly reduced using the Apollo-Chado approach because 1) errors arising from mistakes in data entry on web forms are eliminated, 2) annotations produced by community members are formatted in a way that allows direct loading into the Chado database. Other groups have employed Apollo as part of their community annotation solution. Apollo2Go, a web service adapter developed for the MIPS PlantsDB, allows Apollo clients to obtain GAME-XML-formatted gene evidence from a central database [31]. Our system is more similar to that used by AphidBase, which also allows a direct connection between the Apollo client and the Chado PostgreSQL database [32].

Initially researchers were apprehensive about using Apollo. We held two several-day workshops at Texas A&M University and Iowa State University. Only a small number of researchers (< 40) could attend these workshops. However, the attendees came from many different institutions from around the world, and subsequently held workshops at their institutions. In addition to training sessions, we provided a downloadable tutorial describing annotation approaches that integrated the use of BGD, Ensembl and NCBI. Each of these genome data sources has differences that can hinder the use of multiple sites, particularly in sequence identifiers and vocabulary for the components of genome assemblies. For example, the terms "contig" and "scaffold" are used differently. BGD and BCM-HGSC define the term "scaffold" as an assembly of contigs, and whole "chromosome" as a linear scaffold assembly produced by anchoring scaffolds to a chromosome using a genetic map. However, Ensembl and NCBI use the term "scaffold" for the whole chromosome assembly, and "contig" for the equivalent sequence referred to as "scaffold" at BCM-HGSC. Thus, it was important that we provided the annotators with detailed instructions on using different resources simultaneously. We developed web-based tools that allow users to convert between different coordinate and identifier systems so that researchers can use our website in combination with larger databases (i.e. Ensembl, UCSC Genome Browser, NCBI). For example users can convert a NCBI RefSeq scaffold identifier to a chromosome number based identifier similar to that used at BGD. Users can perform a BLAST search at Ensembl, and receive coordinates on a whole chromosome model (called "scaffold" at Ensembl), and convert those coordinates to a scaffold (as defined by BGD and BCM-HGSC) for annotating with Apollo.

A common desire among users is the ability to view their annotations on a genome browser immediately upon submission. This would be especially useful for long-distance collaborators. This feature was not initially implemented during the BGSAC annotation project, so submitted annotations were not displayed until they were loaded into the Chado PostgreSQL database, which occurred concurrently with the publication of the bovine genome [1]. We have now modified the system to allow direct write-back of annotations to the intermediate (pre-review) Chado database. Connecting the intermediate database to GBrowse then allows immediate viewing of submitted annotations.

Our biggest challenge in the early development of BGD was the need to perform sequence computations (e.g. homolog alignments and consensus gene set) and develop the database and annotation tools simultaneously. However, it was necessary to make our system available prior to completion. We received feedback from users on bugs and we constantly improved the system.

## Conclusions

A goal of BGD is to support annotation of the bovine genome by a widely dispersed research community. We provide tools that supported 147 annotators in contributing 3,871 gene models over a fifteen-week period. These gene models have been integrated into the bovine OGSv2, and may be viewed on individual gene pages at BGD. We continue to encourage contributions from the bovine research community, both in the form of gene models using our annotation system and Wikipedia-style gene discussion pages. Development of BGD is ongoing to accommodate new data types as technologies change.

## Availability and requirements

BGD is publicly accessible at http://BovineGenome.org. Using annotation tools and submitting comments to the wiki require registration. Code under development for BGD is available at http://rubyforge.org/projects/chadoonrails/, http://cgl-gu.svn.sourceforge.net/viewvc/cgl-gu/, and http://prism-api.svn.sourceforge.net/viewvc/prism-api/branches/gu-dev-branch/Prism/app/Prism/.

## List of abbreviations used

BCM-HGSC: Baylor College of Medicine Human Genome Sequencing Center; BGD: Bovine Genome Database; BGSAC: Bovine Sequencing and Analysis Consortium; CCK: Content Construction Kit; CGI: common gateway interface; CSS: cascading style sheets; DAS: Distributed Annotation System; DBI: database interface; EST: expressed sequence tag; GO: Gene Ontology; GFF: generic feature format; GUI: graphical user interface; HTML: hypertext markup language; MVC: model-view controller; NCBI: National Center for Biotechnology Information; OGS: Official Gene Set; ORM: object-relational mapping; QTL: quantitative trait locus (or loci); SNP: single nucleotide polymorphism; SO: Sequence Ontology; UCSC: University of California - Santa Cruz; WYSIWYG: "what you see is what you get"; XML: Extensible Markup Language

## Authors' contributions

JTR installed the Btau_3.1 Chado database and Btau_3.1 genome browsers, and developed the current system that allows annotation write-back to the Chado database and Webstart. JTR and CPC developed the Chado-Apollo connection and installed the Btau_4 database. JTR and CPC developed Chado on Rails and the gene pages. CPC converted the content management system to Drupal. JPS developed the audit tracking system, BGD-specific controlled vocabulary, and automated GO and gene symbol retrieval. CMD developed the annotation portal web pages, CGI scripts for searching annotations, annotation tools, and scripts for processing submitted Chado XML. KLC designed the original web pages, set up the Btau_2 Gbrowse, and created a script to show BLAST hits on GBrowse. DCV modified the code for formatting BLAST output to retrieve data from the Chado database instead of a MySQL database. AV assisted in trouble shooting system compatibility issues in the initial installation of the Btau_3.1 Chado database. CGE is the principal investigator, and performed the computations to create gene evidence tracks and consensus gene sets. All authors reviewed the manuscript and accepted the final version.
